# Increased prevalence of endocervical Mycoplasma and Ureaplasma colonization in infertile women with tubal factor

**DOI:** 10.5935/1518-0557.20190078

**Published:** 2020

**Authors:** Rita CCP Piscopo, Ronney V Guimarães, Joji Ueno, Fabio Ikeda, Zsuzsanna IK Jarmy-Di Bella, Manoel JBC Girão, Marise Samama

**Affiliations:** 1 Clinical Department, Instituto Gera de Medicina Reprodutiva, São Paulo, SP, Brazil; 2 Video-Hysteroscopy Section, Hospital Sírio Libanês, São Paulo, SP, Brazil; 3 Gynecology Department, Universidade Federal de São Paulo – Escola Paulista de Medicina, São Paulo, SP, Brazil

**Keywords:** endocervical bacteria, tubal factor, hysterosalpingography, Ureaplasma urealyticum, Mycoplasma hominis

## Abstract

**Objective:**

Most women suffering from tubal factor infertility do not have a history of pelvic inflammatory disease, but rather have asymptomatic upper genital tract infection. Investigating the impacts of such infections, even in the absence of clinically confirmed pelvic inflammatory disease, is critical to understanding the tubal factor of infertility. The aim of this study was to investigate whether the presence of endocervical bacteria is associated with tubal factors in women screened for infertility.

**Methods:**

This retrospective cross-sectional study involved 245 women undergoing hysterosalpingography (HSG), screened for endocervical colonization by *Chlamydia trachomatis, Neisseria gonorrhea, Ureaplasma urealyticum* and *Mycoplasma hominis*, as part of a routine female infertility investigation between 2016 and 2017.

**Results:**

endocervical bacterial colonization by *Chlamydia trachomatis, Ureaplasma urealiticum, Mycoplasma hominis* and other bacteria corresponded to 3.7%, 9.0%; 5.7% and 9.8%, respectively. There was no colonization by *Neisseria gonorrhea*. The prevalence of tubal factor was significantly higher in patients with positive endocervical bacteria colonization, regardless of bacterial species. When evaluating bacteria species individually, the women who were positive for endocervical *Mycoplasma hominis* had significantly higher rates of tubal factor. Associations between endocervical bacterial colonization and tubal factor infertility were confirmed by multiple regression analysis adjusted for age and duration of infertility.

**Conclusion:**

Besides the higher prevalence of *Mycoplasma* and *Ureaplasma* infectious agents, the findings of this study suggest the possible association of endocervical bacterial colonization - not only *Chlamydia trachomatis* and *Neisseria gonorrhea*, but also *Mycoplasma* species with tubal performance.

## INTRODUCTION

Sexually transmitted infections are a major health issue and are implicated in a number of conditions, including female infertility. Despite the overall importance of genital tract infection still being discussed, tube-peritoneal damage seems to be the foremost way in which infections affect women’s fertility. Pathogenic microorganisms colonizing the lower female genital tract may ascend to the upper genital tract, causing pelvic inflammatory disease (PID) associated with tubal damage, and ultimately to infertility. In the presence of infection, tubal damage may occur in response to adhesions, damage to the tubal mucosa or tubal occlusion impairing oocyte transport (Rhoton-Vlasak, 2000).

Infertility is estimated to affect one in every seven couples in the western world, and one in every four couples in developing countries, with rates of up to 30% in some regions of Africa (Vander Borght & Wyns, 2018). Despite differing estimates concerning the global infertility prevalence, secondary infertility is the most common form of female infertility worldwide, and it is often due to reproductive tract infections with resulting tubal factors (Inhorn & Patrizio, 2015). The risk of infertility is directly proportional to the number of PID episodes, with tubal damage occurring in approximately 15% of the cases. However, most women suffering from tubal factor infertility do not have a history of PID, but rather of asymptomatic upper genital tract infection. Therefore, investigating the impacts of such infections, particularly in the absence of clinically confirmed PID, is critical to understanding the relationship between genital tract infections and tubal factor infertility (Tsevat *et al*., 2017).

A meta-analysis showed that bacterial vaginosis is significantly more prevalent in infertile women and women with tubal factors (van Oostrum *et al*., 2013). Higher prevalence of asymptomatic vaginosis in infertile compared to healthy women has also been recently reported (Babu *et al*., 2017), and the vaginal microbiome is thought to be associated with in vitro fertilization (IVF) outcomes; and therefore, with pregnancy outcomes (Hyman *et al*., 2012).

*Chlamydia trachomatis (C. trachomatis)* infection, a significant global public health issue, is strongly associated with tubal factor infertility and a cause of PID-related morbidity (i.e., infertility and ectopic pregnancy) (Sirota *et al*., 2014). *Neisseria gonorrhoeae( N. gonorrhoeae)* is also known to cause PID, but both infections (*C. trachomatis and N. gonorrhoeae*) may be asymptomatic in some women, and many patients go undiagnosed and untreated (Kreisel *et al*., 2017). *Chlamydia trachomatis, Mycoplasma hominis (M. hominis)* and *Ureaplasma urealyticum (U. urealyticum)* are thought to be opportunistic pathogens in humans, and are often found in the genitourinary tract of healthy women. However, both species have been associated with increased risks of certain pathological conditions, including bacterial vaginosis (Keane *et al*., 2000) and PID (Taylor-Robinson *et al*., 2012).

Except for *C. trachomatis*, there are no reports in the literature confirming the association of asymptomatic endocervical bacteria colonization and tubal factor infertility. This study set out to investigate the association between asymptomatic endocervical bacterial colonization by *C. trachomatis*, *N. gonorrhoeae, M. hominis and U. urealyticum,* and tubal factor infertility in women submitted to initial fertility investigation.

## MATERIALS AND METHODS

### Study design

Retrospective cross-sectional study including women undergoing initial infertility investigation at the *Instituto Gera de Medicina Reprodutiva*, a private reproductive medicine center in São Paulo, Brazil. Procedures in this study are part of routine care at that center. All participants signed an informed consent form and allowed their retrospective data to be used for scientific publication purposes, provided anonymity was respected. Therefore, the study was exempt from approval by the Institutional Review Board.

The medical records of patients seen at the *Instituto Gera de Medicina Reprodutiva* between 2016 and 2017 (n=369) were reviewed, and those reporting both hysterosalpingography (HSG) and endocervical screening for bacterial colonization (n=245) were included in the study. The remaining 124 women were not submitted to HSG due to other classical indications for IVF treatment, such as severe male factor infertility, recurrent miscarriage, a history of salpingectomy or very low ovarian reserve, and were therefore excluded from the analysis.

### Procedures

Selected patients were undergoing their first complete infertility investigation. We collected clinical history, serum hormone, transvaginal ultrasound, hysteroscopy, HSG and endocervical screening data. Endocervical screening for *C. trachomatis, N. gonorrhea, U. urealiticum* and *M. hominis* was requested at the first visit and performed using standard PCR procedures at an outsourced clinical analysis laboratory. Other colonizing bacteria were identified by routine culture of endocervical discharge. All patients were asymptomatic for genital tract infection and results of PCR and the bacterial culture was classified as positive or negative. Patients positive for *C. trachomatis, N. gonorrhea, U. urealiticum* or *M. hominis* were treated with doxycycline (100/mg every 12 hours for 14 days). Infections by other bacteria identified in positive cultures were treated according to results from the antimicrobial susceptibility test.

The 245 women included in the sample were submitted to HSG by a reproductive medicine specialist and classified as having normal or abnormal fallopian tubes. Abnormal tubal findings determined a tubal factor infertility and included any peritubal and/or periovarian adhesions, proximal or distal occlusion or extensive periadnexal adhesions in at least one tube.

### Statistical analysis

We investigated the association between endocervical bacteria colonization and tubal factor infertility. Proportions were presented for categorical data and compared using the Chi-square or the Fisher exact test, as appropriate. Continuous variables were expressed as means and standard deviations (SD) and compared using the Student’s t-test. We made a regression analysis to investigate associations between the presence of endocervical bacteria and tubal factor adjusted for confounders. Statistical analyzes were performed using the SPSS software package (IBM Software Group, USA). We set the level of significance at 5% (*p*≤0.05).

## RESULTS

**Figure 1:**
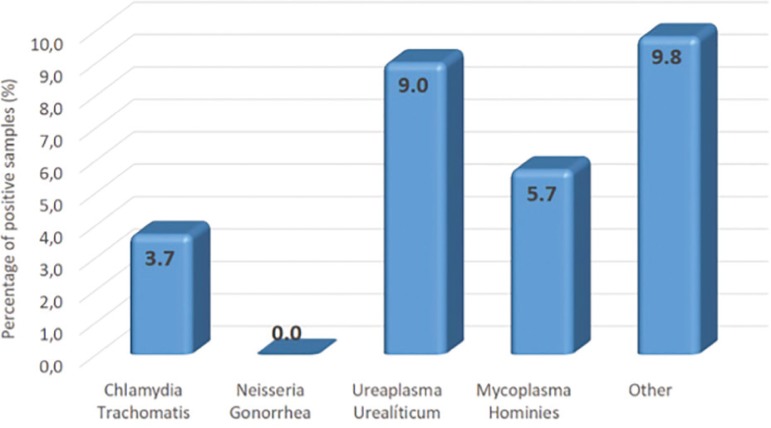
Prevalence of endocervical bacteria in the population studied

**Figure 2:**
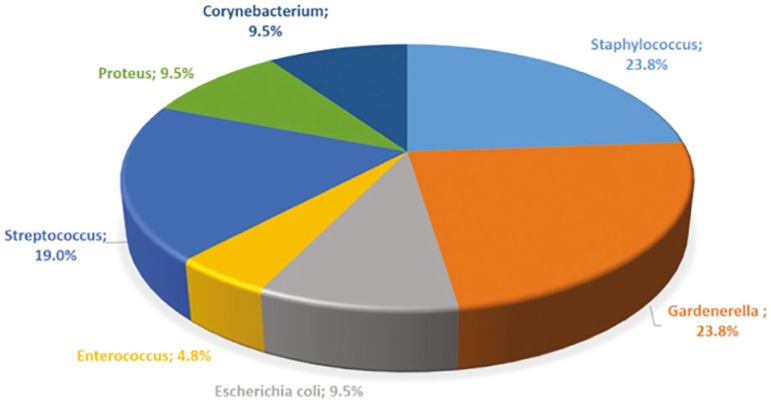
Representative graph of type of bacteria found in the conventional bacterial culture of endocervical samples

The women in this sample had between 22 and 48 years of age (36.3±4.6). Other demographic characteristics were as follows: mean duration of infertility, 4.2 years; mean baseline FSH, 10.2±16.6IU/ml and mean anti-Müllerian hormone levels, 2.6±2.9ng/ml. The overall prevalence of endocervical bacterial colonization was 18.2% and tubal factor infertility was detected in 55.5%. Figure 1 depicts the types of bacteria and respective prevalence rates. *Gardnerella* and *Staphylococcus* (Figure 2) were the most prevalent bacterial genera among other micro-organisms identified in conventional culture of endocervical discharge.

We split the patients into two groups according to the results of bacterial endocervical testing as negative (n=199) or positive (n=46). The demographic characteristics between groups were similar and described in Table 1.

**Table 1:** Demographic characteristics of women presenting negative or positive endocervical bacterial colonization

	Endocervical bacteria colonization		
	Negative	Positive	*p*
N	199	46	
Age (years)	35.8±4.4	35.8±5.6	0.955
Duration of infertility (years)	4.2±2.9	4.1±3.9	0.974
Baseline FSH (IU/ml)	8.2±5.1	6.8±3.2	0.160
Anti-Mullerian hormone (ng/ml)	2.7±3.0	2.5±2.1	0.822

**Figure 3:**
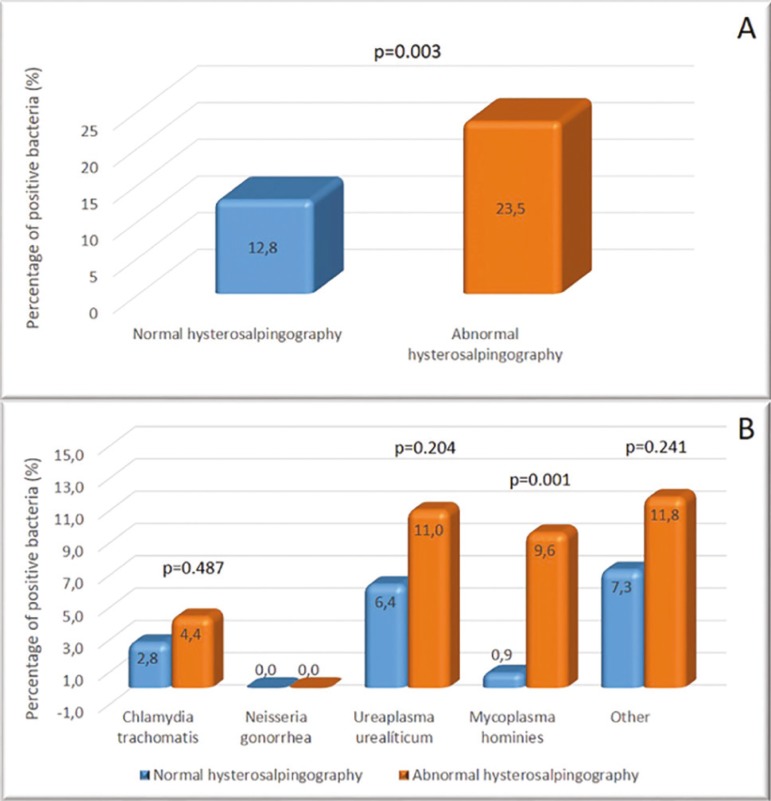
A - Representative graph of the prevalence of endocervical bacteria according to hysterosalpingography findings. B - Representative graph of the prevalence of different endocervical bacteria according to hysterosalpingography findings.

Tubal factor infertility was more prevalent in women with endocervical bacteria colonization, regardless of bacterial species, suggesting an association between endocervical bacteria and tubal factors (Figure 3).

**Figure 4:**
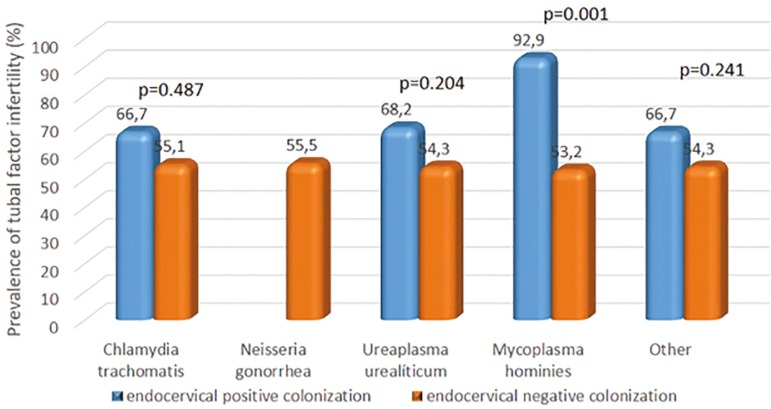
Endocervical colonization. Separate analysis per bacteria also revealed higher percentages of tubal factor infertility when the endocervical positive colonization was present for each bacteria. However, significant results were limited to *Mycoplasma hominis*.

Separate analysis per bacteria also revealed higher percentages of tubal factor infertility when the endocervical colonization was present for each bacteria. However, significant results were limited to *Mycoplasma hominis* (Figure 4). Interestingly, among patients positive for *Mycoplasma hominis*, only one had normal fallopian tubes.

Multiple logistic regression models adjusted for age and duration of infertility were used to confirm associations between endocervical bacterial colonization and tubal factor infertility in this study. Endocervical bacterial colonization was associated with a 2.2-fold increase in the odds of tubal factor infertility (OR: 2.2; *p*=0.028), thereby confirming our findings.

## DISCUSSION

We screened the women in this study for endocervical bacterial colonization as part of an infertility investigation, and 18.2% of the patients tested positive for endocervical bacteria, despite of all being asymptomatic. We found a significant association between tubal factor infertility and endocervical bacterial colonization. However, only *Mycoplasma hominis* was significantly associated with tubal factors following separate analysis per bacteria, despite numerically higher rates of tubal factors in the presence of *Chlamydia trachomatis, Ureaplasma urealyticum* and other bacteria diagnosed by culture of endocervical discharge.

Infection by some of these agents, such as *Chlamydia trachomatis* and *Neisseria gonorrhea*, is known to negatively affect the female reproductive tract. Previous investigations of the Brazilian population revealed comparatively higher prevalence rates of these agents (6.3% and 4.0%; *C. trachomatis*, and *N. gonorrhoeae*, respectively) (Rodrigues *et al*., 2011) than we found in our sample. *Ureaplasma urealyticum* and *Mycoplasma hominis* were the most prevalent bacteria in this sample. This finding is in sync with those from Rodrigues *et al*. (2011) (38.4% and 21.9% prevalence, *Ureaplasma* sp and *M. hominis*, respectively), despite higher percentages reported in that study. In another Brazilian study, the prevalence of *C. trachomatis* was 10.9% and only two cases of *N. gonorrhea* infection were detected in a population of infertile women (Fernandes *et al*., 2014).

Anyway, studies demonstrate that the prevalence of endocervical bacterial infection varies between populations. A Mexican study showed 21.7% and 6.5% of *U. urealyticum* and *M. hominis* prevalence in infertile women, and associated the outcomes with tubal damage in a very small group of patients (Hernández-Marín *et al*., 2016). A Czech study reported 39.6% and 8.1% prevalence of *U. urealyticum* and *M. hominis* in positive endocervical swabs of women undergoing initial fertility testing, respectively (Sleha *et al*., 2016). In contrast, a North American study conducted in New York revealed a 17.2% and 2.1% prevalence of *U. urealyticum* and *M. hominis* in the endocervix at the time of oocyte collection in women undergoing IVF, respectively (Witkin *et al*., 1995). Prevalence rates of 9.0% and 8.6% (*U. urealyticum* and *M. hominis*, respectively) were reported in women of reproductive age in an Italian study (Leli *et al.*, 2018) and, as in this study, *U. urealyticum* was the most common bacteria found in the cervical samples of infertile women in Germany (Graspeuntner *et al*., 2018). These differences in prevalence’s may have reflected disparities in geographical location, medical care (i.e., public or private), population studied and methods for diagnosis used.

A World Health Organization taskforce has been working towards the prevention of tubal infertility for more than two decades, with particular emphasis on the diagnosis of lower genital tract infections. Women infected with *N. gonorrhoeae* and *C. trachomatis* are significantly more prone to bilateral tubal occlusion, despite the lack of pelvic inflammatory disease symptoms (WHO, 1995). Regarding other bacteria, a study screening couples for *Mycoplasma hominis* and *Ureaplasma urealyticum*, found 48% of infertile men and 40% of infertile women positive for *Ureaplasma urealyticum*, with high levels of agreement between positive test results in men and women. Also, lower sperm motility and vitality in *Ureaplasma urealyticum*-positive men suggests negative impacts on male fertility (Lee *et al*., 2013). Similar relationships between *Mycoplasma hominis* and *Ureaplasma urealyticum* colonization and male infertility have been reported elsewhere (Huang *et al*., 2015).

Except for *N. gonorrhoeae* and *C. trachomatis*, the cause-effect relationship of those bacteria colonization and tubal factor infertility are not confirmed. However, endocervical bacterial colonization may lead to vaginal environment imbalances and may be a cofactor in other, more significant infections. The induction of proinflammatory cytokines by abnormal vaginal flora has been associated with bacterial vaginosis. Also, abnormal vaginal flora may furtively invade the uterine cavity, leading to significant endometrial inflammation and immunological changes which, if left untreated, may have serious consequences, including unexplained infertility, tubal obstruction, miscarriage and preterm birth (Viniker, 1999; Spandorfer *et al*., 2001). Bacterial growth has been documented in patients with uterine pathologies such as endometritis, despite the absence of signs of infection (Cicinelli *et al*., 2008). Moreover, IVF outcomes are thought to be affected by the vaginal microbiome on the day of embryo transfer (Hyman *et al*., 2012), and endometrial development towards a proper receptive status, including the establishment of a suitable local immune environment, is influenced by uterine microbiota (Benner *et al*., 2018).

A recent study investigating different sites of the female reproductive tract revealed that distinct bacterial communities form a microbiota continuum from the vagina to the ovaries. The same study also revealed correlations between bacteria detected in the peritoneal fluid and the cervical microbiota, suggesting cervical mucosa sampling may be used to assess endometrial status (Chen *et al*., 2017). Those findings support the association of microbiota in the upper and lower reproductive tracts and then inferred microbial cervical function in uterine-related diseases, in line with association of cervical infection and tubal factor of infertility which was the subject of our study.

Most cases of tubal infertility are actually due to salpingitis, which often results from previous or persistent infections. Bacteria may ascend from the cervical mucosa to the endometrium and fallopian tubes, leading to clinical PID, which in turn is strongly associated with tubal infertility (Ross *et al*., 2018). However, a number of women presenting tubal infertility tend to develop asymptomatic genital tract infections, and therefore do not have a history of PID (Wiesenfeld *et al*., 2012). Then, bacterial vaginosis is thought to be a key factor in upper genital tract disease; still, the link between infection and its respective sequelae, such as infertility, remains to be determined (Graspeuntner *et al*., 2018).

A previous metanalysis showed that bacterial vaginosis is more prevalent in infertile women (van Oostrum *et al*., 2013). Findings from our study suggest endocervical bacterial colonization, particularly by *Mycoplasma hominis* and *Ureaplasma urealyticum*, is associated with tubal factor infertility in asymptomatic infertile women. The regression model employed supported this association, given the two-fold higher odds of tubal factors in women presenting with endocervical bacterial colonization, regardless of bacteria type. However, our study has limitations. First, it is a cross-sectional study and then we may state that there is a possible association between bacterial colonization and tubal factor infertility, but the cause-effect relationship may not be confirmed. Moreover, infertile women with indications for HSG were included in the sample of our study and those with other infertility factors as male infertility, endometriosis, ovarian failure, etc., did not undergo HSG and were not included. In face of that, the prevalence of tubal changes (55%) was higher compared to literature data (up to 30% of tuboperitoneal infertility) (Evers, 2002).

In spite of the evidence of association between Mycoplasma and Ureaplasma infection with infertility (Sleha *et al*., 2016; Witkin *et al*., 1995; Lee *et al*., 2013; van Oostrum *et al*., 2013; Graspeuntner *et al*., 2018) and spontaneous preterm birth resulting from induced inflammation (Murtha & Edwards, 2014), the European STI Guidelines Editorial Board does not recommend routine testing or treatment for asymptomatic or symptomatic male and female patients with *M. hominis, U. urealyticum* or *U. parvum* (Horner *et al*., 2018). A consensus as to whether or not such infections should always be treated is lacking and there are controversies regarding the classification of Mycoplasma species as pathogenic and worthy of treatment or part of the non-pathogenic bacterial flora (Patel & Nyirjesy, 2010).

To the authors’ knowledge, this study is the first to investigate the prevalence of *Chlamydia trachomatis, Neisseria gonorrhea*, *Ureaplasma urealyticum* and *Mycoplasma hominis* evaluated by PCR, in endocervical samples of infertile women and their potential association with HSG findings. The current diagnostic workflow for female infertility comprises clinical history analysis and serological screening for previous sexually transmitted infections (STI), as well as the investigation of reproductive tract abnormalities. Findings of this study suggest the possible association of endocervical bacterial colonization - not only for *Chlamydia trachomatis* and *Neisseria gonorrhea,* but also for *Mycoplasma* species such as *Ureaplasma urealyticum* and *Mycoplasma hominis* - with tubal functioning. The latter organisms are not the focus of investigation in the routine infertility clinics, but ours and other studies demonstrated their high prevalence and having been associated with higher odds of tubal factor of infertility in this sample. Other studies should be developed to confirm the cause-effect relationship between *Ureaplasma urealyticum* and *Mycoplasma hominis* endocervical infection and tubal factor infertility.
